# Changes in the physiological activity of parenchyma cells in *Dalbergia odorifera* xylem and its relationship with heartwood formation

**DOI:** 10.1186/s12870-023-04592-2

**Published:** 2023-11-14

**Authors:** Ruoke Ma, Jia Luo, Weijie Wang, Yunlin Fu

**Affiliations:** Key Laboratory of National Forestry and Grassland Administration for Fast‑Growing Tree Breeding and Cultivation in Central and Southern China, College of Forestry, Guangxi University, Nanning, 530004 China

**Keywords:** Heartwood formation, *Dalbergia Odorifera*, Parenchyma cells, Transition zone, Water content, Secondary metabolites

## Abstract

**Background:**

The formation of a tree’s heartwood gives the wood properties such as natural decay resistance and aesthetic color, and often directly determines the value of wood products. Regulating the quantity and quality of heartwood is of great importance to the use of wood. However, the mechanism of heartwood formation has been poorly understood.

**Results:**

Using *Dalbergia odorifera* as the study species, the number of starch grains, the morphology of the nuclei, the changes in the content of water and secondary metabolites were observed continuously in the radial direction of the xylem. The results show that from the outer toward inner sapwood, the starch grains are abundant, the length to diameter ratio of the nuclei is decreasing, and the morphology changes from elongated elliptical and then to round. In the outer transition zone, the starch grains begin to decrease abruptly and the nuclei shrink at a slower rate, with a radial width of approximately 2 mm. In the inner transition zone, the heartwood color begins to appear, the starch grains disappear and a few nuclei with reduced fluorescence are present, with a radial width of approximately 1 mm. Heartwood formation after complete disappearance of the nuclei. The moisture content of the heartwood is higher than that of the sapwood, and the inner transition zone is where the content rises. The secondary metabolites of the heartwood begin to accumulate in large quantities in the inner transition zone.

**Conclusion:**

Based on the physiological changes of parenchyma cells in the xylem, the radial width of the transition zone of *Dalbergia odorifera* is clearly defined as approximately 3 mm. Both the water and secondary metabolite abrupt changes occur at the final stage of programmed cell death, and neither is a direct cause of programmed cell death in parenchyma cells.

**Supplementary Information:**

The online version contains supplementary material available at 10.1186/s12870-023-04592-2.

## Background

Sapwood is located in the outer layer of the tree xylem and contains living cells and nutrients (e.g. starch). When the sapwood transforms into heartwood, the intracellular nutrients are converted into secondary heartwood metabolites, the parenchyma cells are inactivated and the color of the xylem in some tree species subsequently darkens [1, 2]. The transition zone is the area where the sapwood develops and transforms into heartwood and varies in width between tree species, usually containing one to two growth rings [3, 4]. The ultramicroscopic morphological and structural changes that occur in the xylem parenchyma cells in the transition zone play an integral role in the formation of heartwood [5, 6]. In previous studies, the sapwood-transition zone-heartwood distinction was usually based on xylem color and position, which is highly subjective and tends to ignore the influence of changes in the physiological function of parenchyma cells on heartwood formation. Typical physiological events that occur in parenchyma cells from sapwood toward transition zone include the shrinking and disappearance of the nuclei and nucleolus, the continuous depletion of nutrients such as starch granules and lipids, the rupture of the vacuoles and disappearance of the cell contents [3, 7, 8]. Sorting out the physiological changes during parenchyma cell development and identifying the location of the transition zone is a prerequisite for the study of heartwood formation.

Heartwood formation is a developmental process in which xylem parenchyma cells are regulated by a combination of endogenous gene expression and exogenous environmental factors [9, 10], culminating in the programmed death of parenchyma cells and the biosynthesis of heartwood secondary metabolites. There are multiple perspectives on the environmental factors responsible for programmed cell death in parenchyma cells. Parenchyma cells biosynthesis secondary metabolites in the transition zone, and it has been suggested that the phenolics in these may be toxic to the cells causing parenchyma death, However, there is no direct experimental confirmation [11]. In addition, the moisture content of the xylem sapwood and heartwood usually changes after heartwood formation. In coniferous wood, the moisture content of the transition zone is lower than that of the sapwood, and it is speculated that the lack of moisture may have caused the death of the parenchyma cells [12]. Overall, there is still no uniform understanding of the causes of programmed death in parenchyma cell.

*Dalbergia odorifera* T. Chen (*D. odorifera*), commonly known as Hainan Huanghuali, is native to Hainan and has been widely introduced to Guangxi, Guangdong, Fujian and Yunnan in China [13]. In addition, the heartwood of *D. odorifera* trunk and roots is a valuable traditional Chinese medicine named “Jiangxiang”, which has various biological activities such as anti-tumour, anti-inflammatory, antioxidant, anti-bacterial, and anti-thrombotic, and is widely used in herbal preparations for the treatment of blood disorders, anaemia, swelling, necrosis and rheumatism [14]. The market demand for *D. odorifera* is large, with approximately 250–300 tons of raw material required annually for the traditional Chinese medicine market [15]. In recent years there have been large plantations and the plantation area has exceeded 3,500 hectares, but the formation of heartwood is slow and there is currently no available heartwood. Effective regulation of heartwood formation is the key to solving the current problems in the development of the *D. odorifera* forest industry.

The parenchyma cells undergo physiological events such as programmed death, changes in moisture content and biosynthesis of secondary metabolites in the transition zone. To clarify the patterns of changes in physiological events and their relevance to heartwood formation, *D. odorifera* was used as the study species to clarify the location of the transition zone by observing in situ the patterns of morphological and quantitative changes in starch grains and nuclei occurring in parenchyma cells throughout the xylem radially; Determining changes in xylem moisture and content of characteristic metabolite components to investigate their relationship with programmed cell death in parenchyma cells.

## Results

### Morphological and quantitative changes in starch grains

**Fig. 1:**
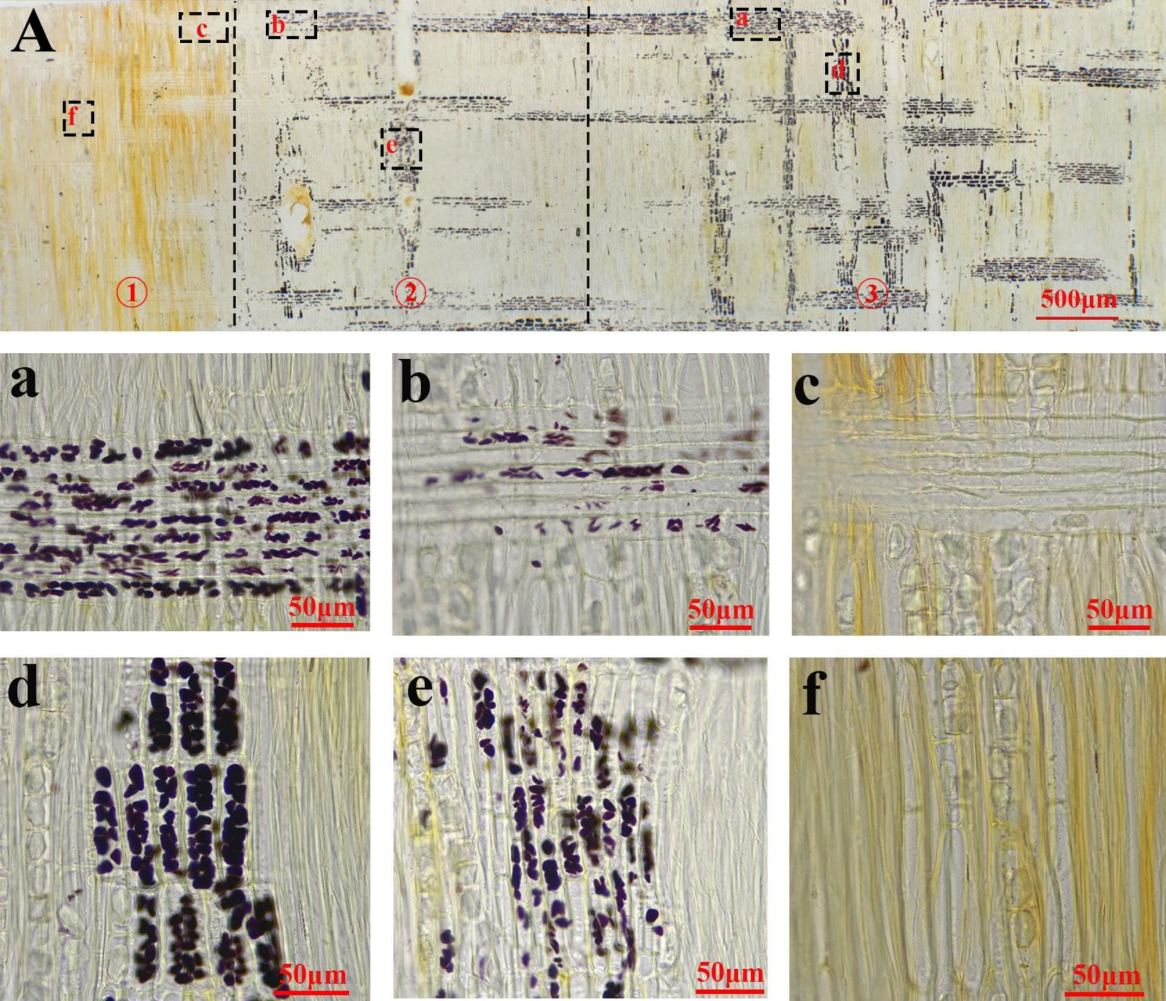
Radial sections showing the presence and absence of starch grains in parenchyma cells. **A** shows the overall variation in the content of starch grains in sapwood-transition zone-heartwood. **a-c** are ray parenchyma cells and **d-f** are axial parenchyma cells. Sections are divided into three areas, ①is the area of heartwood color appears, ②is the area of starch grain reduction and ③ is the sapwood

**Fig. 2:**
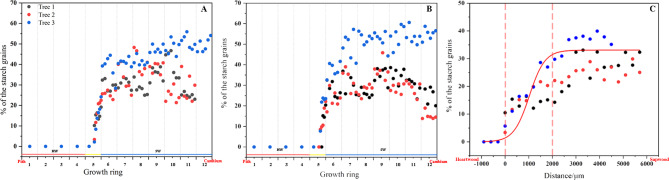
Radial sections showing the presence and absence of starch grains in parenchyma cells. **A** shows the variation of starch grain content in per unit area of ray parenchyma cells and **B** shows the variation of starch grain content per unit area of axial parenchyma cells. The numbers 1–12 correspond to the different growth rings of the xylem, with heartwood in 1-4th growth rings, transition zone in 5th growth ring and sapwood in 6-12th growth rings. **C** depicts the distance between the change in starch grain content and the appearance of heartwood color in a part of the whole xylem, where the heartwood color starts to appear at x = 0 and x = 2000 μm is where the starch grain content decreases abruptly, and the curve is the trend of starch grain change obtained by using the Boltzmann fitting method. The positive axis is in the direction of the cambium and the negative axis is in the direction of the heartwood

Figure 1 shows the variation of starch grains in the ray and axial parenchyma cells of *D. odorifera* xylem from the sapwood to the heartwood. The starch grains are present in the ray and axial parenchyma cells and are stained with I_2_-KI to form brownish-black round or elliptical granules in the outer to inner sapwood of the xylem (growth ring 6–12). The ray parenchyma cells (Fig. 1, a) and axial parenchyma cells (Fig. 1, d) are physiologically and metabolically active and contain large amounts of starch grains that occupy almost the entire cell lumen. The trend in the number of starch granules was quantified by the proportion of the projected area of starch granules in a radial section per unit area (Fig. 2). The content of starch grains fluctuated slightly at different locations in the sapwood, but the content of starch grains within the parenchyma cells was at a high level (Fig. 2, A, B). The proportion of starch grains in the parenchyma cells in the transition zone (at the 5th growth ring) decreases rapidly (Fig. 2, A, B). After the appearance of heartwood color (Fig. 1, A, ①), the starch grains almost disappear, except for the presence of scattered starch grains (Fig. 1, c, f). The proportion of the area of starch grains reduced inward from at approximately 2000 μm outer than the color-boundary between transiton zone and sapwood (Fig. 2, C). Area 2 in Fig. 1A is the outer transition zone. After heartwood formation, the starch grains in the parenchyma cells of the sapwood disappear completely (Fig. 2A, B).

### Changes in the morphology and number of nuclei

**Fig. 3:**
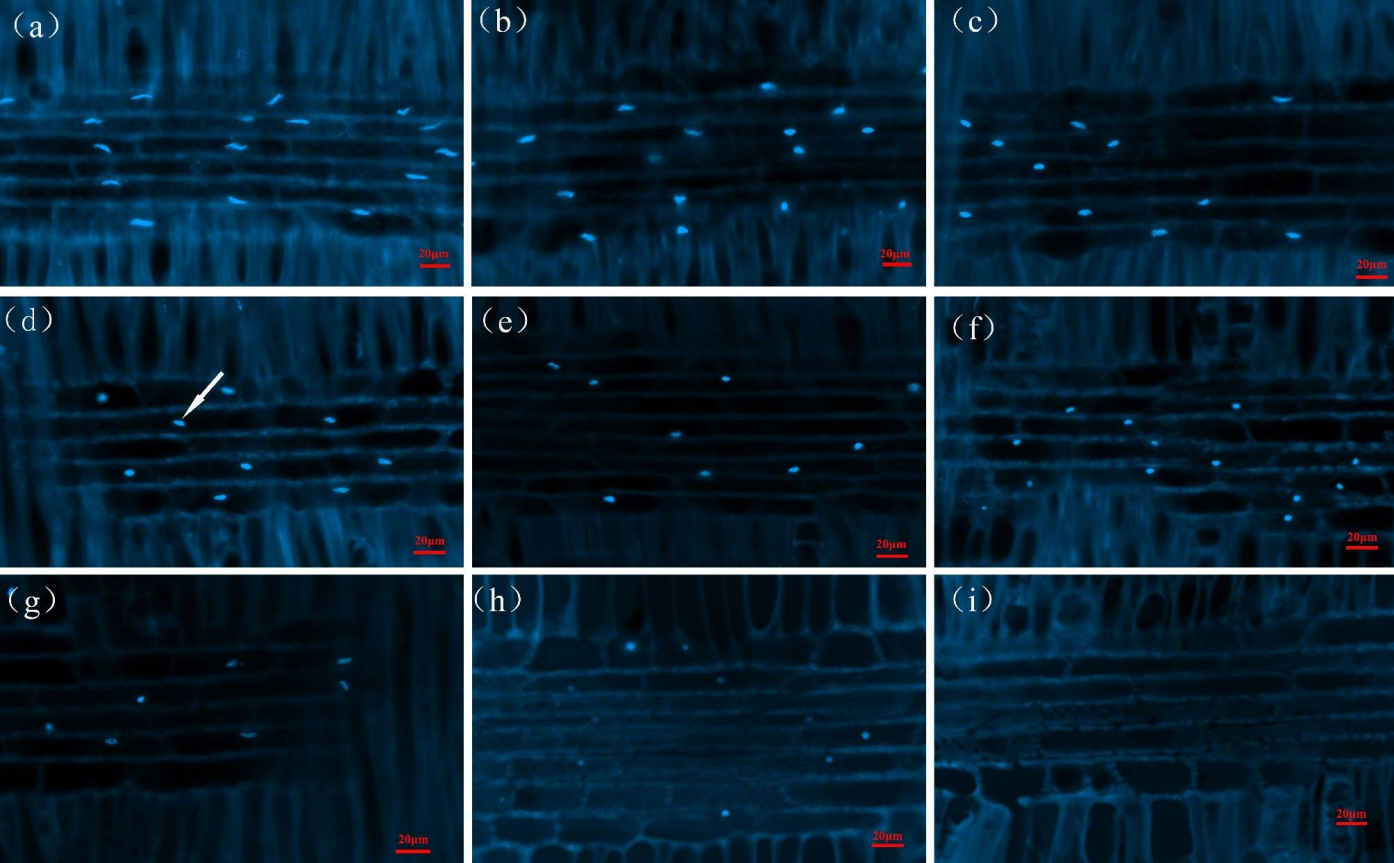
Morphological changes in the staining of nuclei at different positions in the xylem. **a-f** are nuclei in parenchyma cells at different locations in the sapwood, **g** and **h** are locations in the transition zone, **i** is the location in the heartwood, and the nuclei are indicated by the arrows in figure d

**Fig. 4:**
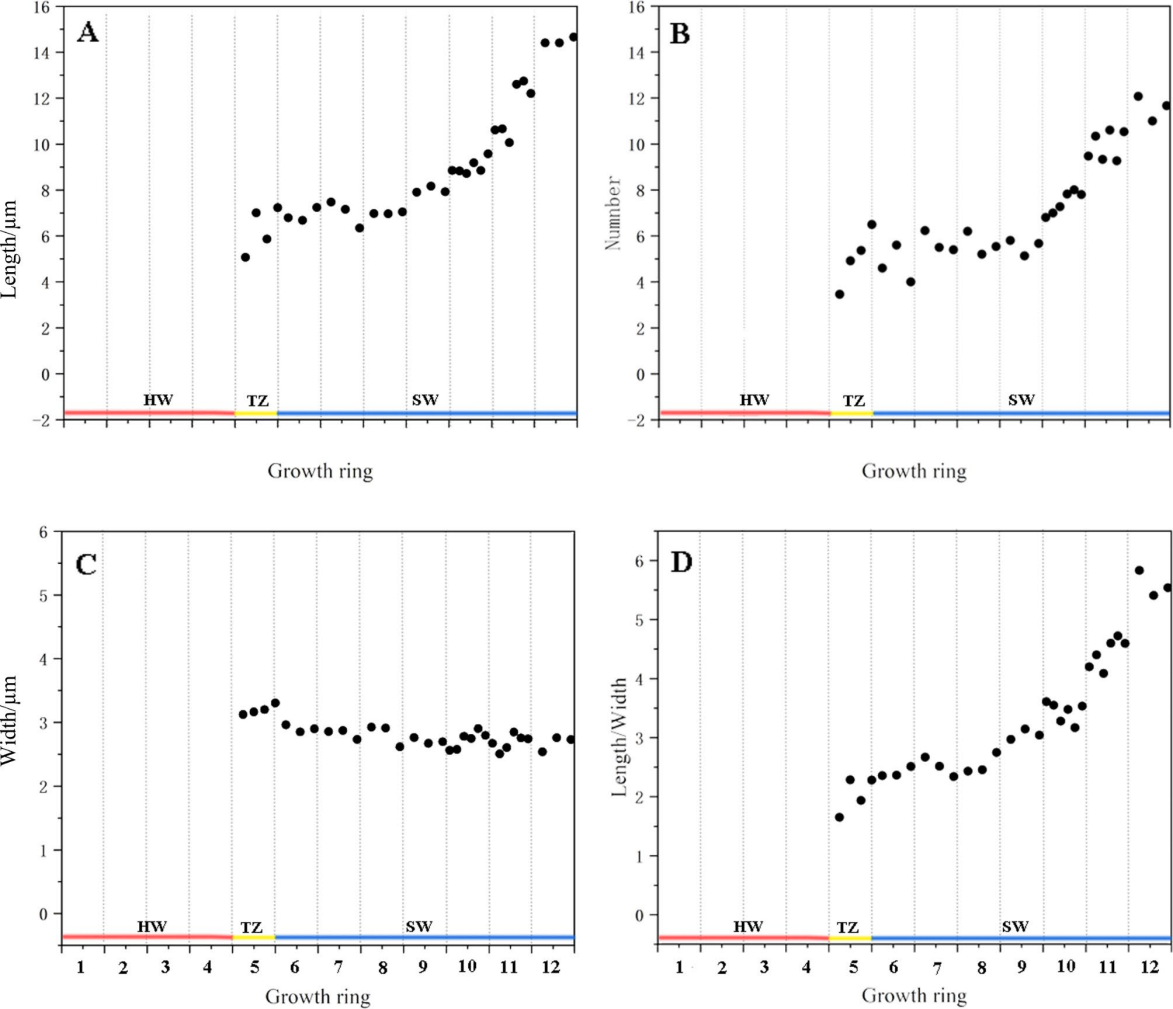
Statistics of morphological indicators of nuclei in different parts of the xylem. **A**: the variation in length of nuclei; **B**: the variation in number of nuclei; **C**: the variation in width of nuclei; **D** shows the variation in length to diameter ratio of nuclei. The results are only from ray parenchyma

Figure 3 shows the morphological changes of the nuclei in the ray parenchyma cells at various locations in the xylem after DAPI staining. Figure 3B shows the results of counting the morphological indicators (length, width and length-diameter ratio) and number of nuclei per unit area. The nuclei of the parenchyma cells in the outer sapwood were mostly fusiform or elliptical and close to the center of the cell lumen (Fig. 3, a-f). From the outer to the inner sapwood, the nuclei gradually became shorter in length (Fig. 4A), the width increases slightly (Fig. 4C), and the length to diameter ratio and number tended to decrease (Fig. 4B and D). Upon entering the transition zone, the nuclei of some parenchyma cells disappeared (Fig. 3g). Unlike the pattern of change in the number of starch grains, the nuclei did not disappear completely immediately after the appearance heartwood color, but the fluorescence response became weaker and the nuclei shrinks and gradually approached round in morphology (Fig. 3h), a characteristic change of about 1 mm in radial width in the xylem (Figure S1). The disappearance of the nuclei in the ray parenchyma cells marked a complete loss of cell viability. The overall pattern of change in nucleus morphology from sapwood-transition zone-heartwood was: fusiform (elliptical)-rounded - fluorescence became weaker-disappeared, and the length to diameter ratio also showed a general trend of gradually decreasing from sapwood to heartwood. Based on the number of starch grains and changes in nucleus morphology in parenchyma cells, the width of the transition zone in the radial direction of the xylem was determined to range from approximately 3 mm.

### Distribution of moisture content in the xylem

**Fig. 5:**
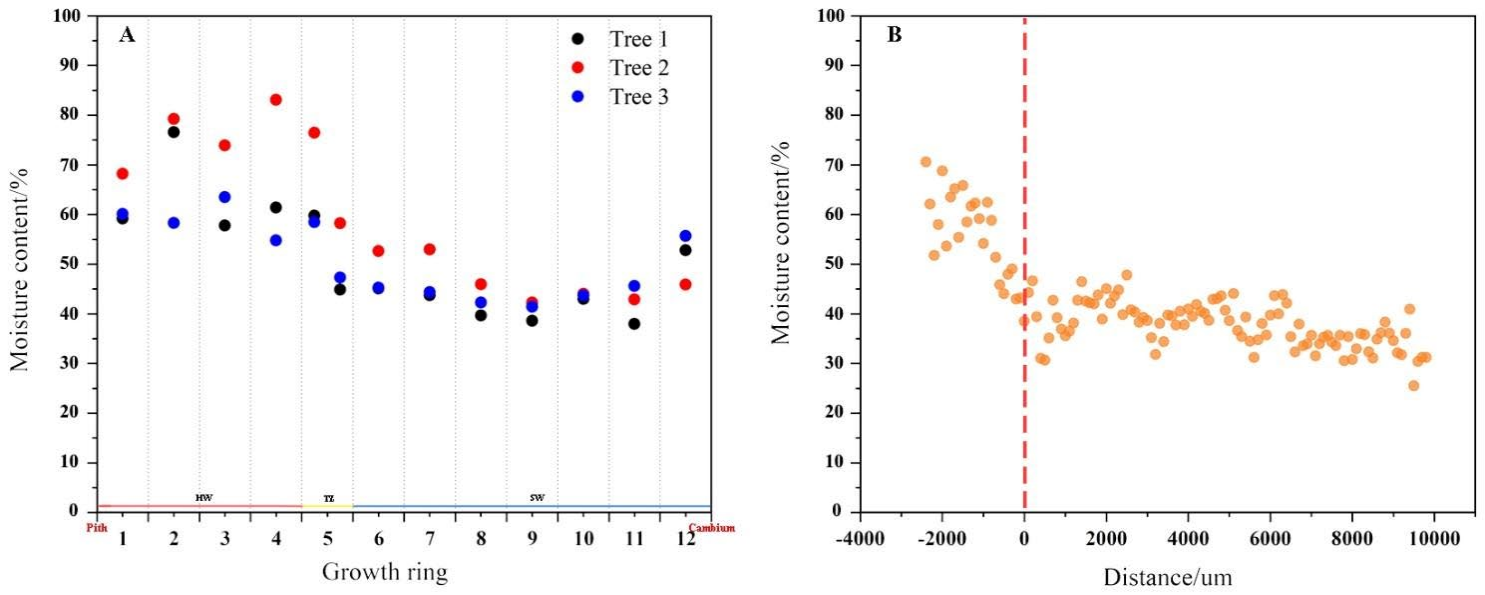
Variation in water content in different areas of the xylem. **A** show the moisture content of the different growth rings (1–12) in the xylem. **B** shows the variation of heartwood moisture with distance from the heartwood, using the position where the heartwood color starts to appear as point 0

In order to characterise the distribution of moisture in the xylem as accurately as possible, vacuum freeze drying is used to measure the moisture content, which effectively avoids the evaporation of low boiling point volatile metabolite components from the xylem by high temperature drying methods, and to determine the true moisture content as far as possible. Figure 5 A shows the distribution of moisture content in the different growth rings of the xylem, with the sapwood (growth rings 6–12) having a moisture content of approximately 45% (dry weight) and the heartwood (growth rings 1–4) having a higher moisture content than the sapwood at approximately 63%, with significant changes in moisture content occurring in the fifth growth rings. Overall, the moisture content of the sapwood of *D. odorifera* was significantly lower than that of the heartwood, which is consistent with the results of previous studies and differs from the results of studies where the moisture content of the heartwood of coniferous woods such as Japanese larch (*Larix kaempferi*) was lower than that of the sapwood [16]. Figure 5B illustrates the position of the relationship between moisture content and heartwood formation. The position of the change in moisture content is approximately 1 mm in width after the disappearance of the starch grains and after the appearance of the heartwood color. The moisture content fluctuates in the radial direction of the xylem and correlates with the cell type and structure of the xylem at the measurement location.

### Distribution of characteristic heartwood extractive in the xylem

**Fig. 6:**
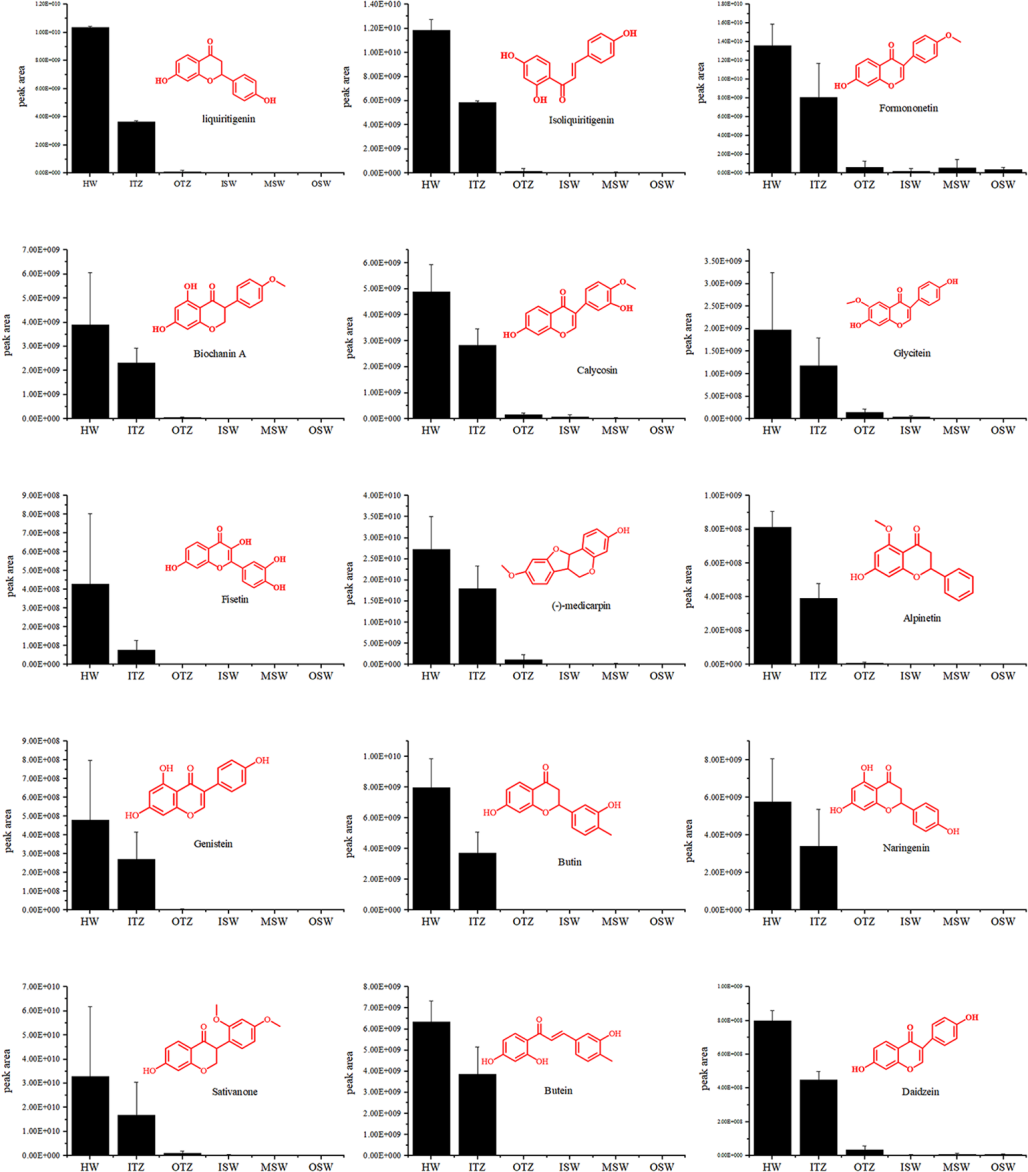
Distribution of the content of characteristic metabolites in different parts of the xylem. HW is the heartwood area, ITZ and OTZ are the inner and outer transition zones, ITZ is the area after the heartwood color has been generated and is approximately 1 mm wide, OTZ is the transition zone where the heartwood color has not changed and is approximately 2 mm wide, ISW is the inner sapwood layer, MSW is the middle sapwood position and OSW is the outer sapwood area. Bars represent ± SD. Biology repeats three times

The color of *D. odorifera* sapwood is pale yellow and changes to dark brown when the heartwood is formed and collects a large amount of heartwood extractive components [17]. The flavonoids, which are characteristic heartwood compositions (secondary metabolites of the heartwood), are key compositions in the color formation of the wood. The content of the characteristic heartwood compositions was analysed by UPLC-MS (Fig. 6). Identification of the flavonoid components was based on retention time, information on the cleavage of secondary fragments of the compositions and comparison with standards, and relative content was measured by integrating the signal intensity of the mass spectra. 15 characteristic flavonoid compositions were highest in the xylem of the heartwood, with the transition zone being the location where the heartwood compositions begin to accumulate in large quantities. Most of the secondary metabolites were low or undetected in the outer transition zone and sapwood. Some of the heartwood signature compositions could be detected in the outer transition zone (e.g. formononetin, calycosin, medicarpin, and daidzein), and formononetin was detected n all regions of the xylem. The distribution of the different secondary metabolites in the xylem can vary slightly, but the pattern of results is consistent in that the content of the heartwood compositions rises abruptly in the transition zone.

## Discussion

### Location of xylem regions based on physiological changes in parenchyma cells

Xylem parenchyma cells play an irreplaceable role in heartwood formation by changing from living cells that maintain protoplasm along with physiological activity to dead cells [6]. In this study, we first use the changes in morphology of parenchyma cells as a clue to map out the main course of programmed cell death. As one of the main forms of energy storage in parenchyma cells, starch grains provide the energy and material basis for the cells, and therefore the number of starch grains can reflect the activity of the cells to a certain extent [5].

Large numbers of starch grains are present in the parenchyma cells of *D. odorifera* sapwood (6th-12th growth ring), and after heartwood formation (1st-4th growth rings), the starch grains disappear and their number decreases in a very narrow range of locations within one growth ring (5th growth rings). Ring 5 is the place where cytological and physiological changes occur in parenchyma cells and surrounding wood from sapwood to heartwood, i.e., transition zone. This is because (a) in ring 5, starch degrading (disappearing of starch grain) is pronounced (Fig. 2A and B); (b) moisture content differed between the inner part and outer part of ring 5 (Fig. 5A); (c) nuclei can be observed in ring 5 but not in ring 4 (Fig. 4); (d) the amounts of secondary metabolites (or heartwood substances) differed between the inner part and outer part; The starch grains almost disappear after the color of the heartwood appears. About 2000 μm before the onset of heartwood color is the point at which the number of starch grains begins to decrease sharply. The morphology of the nuclei is also often used as an important indicator to characterize the viability of the cells [18, 19]. From cambium to pith, the morphological changes in the nuclei of the parenchyma cells of the xylem of *D. odorifera* roughly go through three stages. In the first stage the morphology of the nuclei changes from long spindly to elliptical, the length of the nuclei gradually shortens, the width slightly increases, the length to diameter ratio gradually becomes smaller, the location where this process exists is mainly from the outer to the middle sapwood (1st-4th growth rings), in addition, the number of nuclei per unit area in this stage gradually decreases and the cell shrinks. The second stage is characterised by a morphologically elliptical nuclei with insignificant changes in number and volume. The third stage is a radial range lasting approximately 1 mm after the appearance of the of heartwood color, a stage in which the fluorescence response of the nuclei becomes weaker, for reasons that may be related to the loss of chromatin or fragmentation of DNA [20, 21]. The nuclei eventually disappear after heartwood formation, and the same pattern is found in coniferous species [19]. The complete disappearance of the nucleus follows the disappearance of the amyloplasts.

The microscopic changes in the morphology and structure of parenchyma cells in the transition zone are closely related to the formation of heartwood. Based on the pattern of changes in starch grains and nuclei, the xylem region was divided into six positions: outer sapwood (long spindly to elliptical nuclei) - inner sapwood (smaller nuclei length to diameter ratio) - outer transition zone (~ 2 mm width, reduced starch grain content) - inner transition zone (~ 1 mm, weakened nuclei fluorescence response, starch grains disappear) - heartwood. The transition zone is within a narrow zone of growth ring, with a radial width range of ~ 3 mm.

### Relationship between programmed death of parenchyma cells and moisture and secondary metabolites

After heartwood formation, secondary metabolites are biosynthesised by parenchyma cells and dispersed through the pit to other surrounding tissues, altering the original structure and properties of the xylem cell walls and subsequently affecting or changing the overall distribution of moisture in the xylem. In a study of moisture in the xylem of coniferous species (*Larix kaempferi* and *Cryptomeria japonica*), it was found that the moisture content of the heartwood region was lower than that of the sapwood, with the beginning of the decrease being in the transition zone, presumably due to the programmed death of parenchyma cells as a result of the lack of moisture [12, 22] However, in the present study it was found that the average moisture content of *D. odorifera* heartwood was much higher than that of the sapwood, and that the location where the change occurred was in the transition zone, where the moisture content tended to equilibrate dynamically after heartwood formation, similar findings were found for *Eucalyptus cloeziana* (sapwood moisture content ca. 79%, heartwood ca. 95%, unpublished data) and *Manglietia glauca* [23] were found to be similar. This indicates that the wood properties of coniferous and hardwood are somewhat different, and that the location of the moisture change occurs at the last stage (transition zone) of programmed cell death in the parenchyma cells, suggesting that moisture deficiency is not a direct cause of programmed cell death in *D. odorifera*.

The heartwood of *D. odorifera* is rich in secondary metabolites, dominated by flavonoids. Flavonoid compositions are often considered to be the main factor influencing the formation of heartwood color and have therefore received widespread attention [24]. Secondary metabolites have biological activities such as antibacterial activity and are effective in preventing xylem invasion by foreign organisms such as fungi. To analyse the relationship between secondary metabolite biosynthesis and programmed death of parenchyma cells, the location of secondary metabolite biosynthesis and changes in parenchyma cell activity in the xylem were compared. The flavonoid components characteristic of *D. odorifera* are extremely low in the outer layer from the sapwood to the transition zone, with significant aggregation of secondary metabolites following the disappearance of starch grains in the parenchyma cells (the inner transition zone). The 15 characteristic flavonoids of *D. odorifera*, including isoliquiritigenin, daidzein and genistein, showed essentially the same pattern. There are two possible reasons for this phenomenon, one being that secondary metabolites are synthesised in small amounts in the sapwood and then transported to the inner xylem through transverse channels such as ray parenchyma cells, if this occurs, the content of secondary metabolites should tend to increase in a gradient in the radial direction in the xylem, but no similar phenomenon occurs in the distribution of the selected characteristic compositions and most of them are not detected in the sapwood. Secondly, secondary metabolites are biosynthesised in situ from precursor material in the transition zone and reach their highest levels in the heartwood, the distribution of *D. odorifera* flavonoids being more in line with the second scenario. Aggregation of secondary metabolites occurs in the final stages of programmed cell death after the activity of the parenchyma cells in the heartwood has been reduced, and is accompanied by the process of programmed cell death and the continuous production of aggregates. It is assumed that the biosynthesis of secondary metabolites is not the direct cause of the death of *D. odorifera* parenchyma cells, but rather a metabolic manifestation of the senescent death of parenchyma cells.

## Conclusion

The pattern of changes in parenchyma cell starch grains and nuclei in the radial direction of the xylem suggests that the physiological metabolic activity of parenchyma cells decreases continuously during heartwood formation. The xylem zones are distinguished according to the sequence of key physiological events in the development of parenchyma cells from development to programmed death: nuclei are long and spindle-shaped (outer sapwood) - nuclei become smaller in aspect ratio (middle and inner sapwood) - starch grain content is reduced (~ 2 mm width, outer transition zone) - heartwood color begins to develop and a few live cells are present (~ 1 mm, inner transition zone) - Death of parenchyma cells and complete formation of heartwood color (heartwood), the extent of the *D. odorifera* transition zone is very narrow, within an growth rings, ~ 3 mm. increased moisture content and accumulation of secondary metabolites are found in the inner transition zone, both of which are not directly responsible for the programmed cell death of parenchyma cells.

## Materials and methods

### Plant materials

**Fig. 7:**
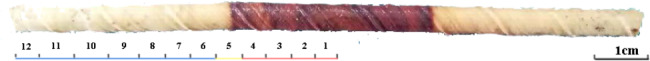
Diagram of the core of *D.odorifera*;The numbers 1–12 are the individual growth rings of the xylem

Three 12-year-old *D. odorifera* trees with the generally consistent growth conditions, were collected at nursery stock base of Guangxi University in Nanning, Guangxi Zhuang Autonomous Region, China, in June 2021. The tree height was 7.5, 7.3 and 9.9 m and the diameter at breast height is 14.5, 14.3 and 13.7 cm (1.2 m above ground). The increment cores (Fig. 7) were drilled separately in the north-south direction before felling and then quickly placed in 4% paraformaldehyde phosphate fixative for observation of nuclei and starch grains. Trees were felled and sawn into discs (~ 2 cm thickness) with a chainsaw, and the discs were quickly trimmed into long strips over the pith. Samples were submerged in liquid nitrogen and transported on dry ice to the laboratory for storage in -80 °C refrigerators for moisture and secondary metabolite detection.

### Staining observations and relative content of starch grains and nuclei

The cores were soaked in the fixing solution for more than 48 h and then divided into small sections from the outer sapwood (near the cambium) to the heartwood according to the annual rings. Wood segments were fixed to the carrier table with freezing solution, and radial section samples were obtained using a cryomicrotome (Leica CM1860, Germany) with a freezer temperature of -20 °C.

To observe changes in the relative content of starch grains, radial Sect. (15 μm thickness) were stained with 1% (w/v) I_2_-KI solution for 20 min, dehydrated according to a gradient of 30-50%-70-100% ethanol solution, resin-sealed and then observed under a light microscope and photographed. The relative content of starch grains was counted: five randomly selected ray parenchyma cell regions of 250 × 100 μm (length × width) or axial parenchyma cell regions of 50 × 200 μm for each sample, and the area of starch grains was measured using ImageJ software. The fixed areas for nuclei were selected to be containing only the targeted parenchyma cells. The changes in starch grains were expressed as the proportion of starch grains per unit area, and the biological replicates 3 times.

To observe changes in the morphology and number of nuclei, radial Sect. (10 μm thickness) were placed in DAPI (4’,6-diamidino-2-phenylindole) solution, protected from light for 15 min, wash 3 times with phosphate buffer for 5 min each, sealed with an anti-fluorescence quencher and then observed under a fluorescent microscope and photographed. Five areas (250 × 100 μm) including only ray parenchyma cell were randomly selected for each sample to count the number of nuclei. The fixed areas for nuclei were selected to be containing only the ray parenchyma cells. Five nuclei per region were randomly selected and their length and width were determined using ImageJ software. The biological replicates 3 times and the final average was taken.

### Determination of moisture content

Ultra-low temperature frozen (-80 °C) wood samples (radial × tangential × axial approx.16 × 2 × 15 mm) were cut into string sections (thickness 50 μm, 2 sections per group) using a frozen slicer, after which the sections were placed in pre-weighed centrifuge tubes (accuracy 0.0001 g) in the freezer of the slicer (freezing temperature − 20 °C). After weighing (W0), the slices were weighed (W1) in a vacuum freeze dryer with the lid open for more than 10 h, until the slices were absolutely dry. The formula for calculating the water content: xylem water content (%) = (W0-W1)/W1 × 100, where W0 and W1 are the mass of the sample before and after drying respectively.

### Determination of characteristic heartwood components

After freezing in liquid nitrogen, the blocks were freeze-dried for 24 h and then trimmed into small strips of approximately 20 × 1.5 × 15 mm (radial × tangential × axial, after which a sledge microtome was used to obtain a tangential section (set slice thickness 25 μm). The strip includes the heartwood, inner transition zone, outer transition zone, and the inner sapwood. In addition, samples from the middle sapwood are approximately 1.5 cm before the color of the heartwood appears and samples from the outer sapwood are approximately 3 cm before the color of the heartwood appears. The samples were extracted by ice-water sonication for 30 min (40 kHz) at a ratio of sample (weight): extractant = 1 mg:100 µL, with methanol: water = 4:1; afterwards, the samples and solvent mixtures were statically extracted for 24 h in a 4 °C refrigerator, during which time the samples were sonicated twice in ice water for 30 min each time. 13,000 rpm, centrifuged at 4 °C for 15 min and the supernatant was extracted. Finally, the sample solution was stored in a liquid phase injection vial for subsequent analysis.

The relative contents of medicarpin, formononetin, calycosin, butein, isoliquiritigenin, liquiritigenin, butin, glycitein, naringenin, genistein, alpinetin, daidzein, biochanin A, fisetin and sativanone in different parts of the xylem were determined by ultra performance liquid chromatography-mass spectrometry (UPLC-MS) (Thermo Fisher Scientific, USA). The relative contents of 15 characteristic metabolites and the identification of metabolites were done against standards [17]. UPLC conditions: the chromatographic column type was ACQUITY UPLCBEHC18 column (1.7 μm × 2.1 mm×50 mm); mobile phase: A was an aqueous solution containing 0.1% formic acid and B was methanol. The mobile phase A was 0.1% formic acid in water and B was methanol; gradient elution, 0 ~ 15 min, 5%~80% B; 15.0 ~ 18.0 min, 80%~100% B; 18.0 ~ 18.1, 100 ~ 5% B, 18.1 ~ 21 min, 5% A; flow rate was 0.3 mL-min-1, column temperature was 30 °C, injection volume was 1 µL. High resolution mass spectrometry conditions: ESI ion source at 3.0 kV, temperature 300 °C; transfer capillary temperature 320 °C; sheath gas 206 KPa; auxiliary gas flow rate 69 kPa; scan modes Full MS and Full MS/dd-MS2, mass range 100–1000 Da, collision voltage settings 30,40 and 80v.

### Electronic supplementary material

Below is the link to the electronic supplementary material.

Supplementary Material 1

## Data Availability

All data generated or analysed during this study are included in this published article.
