# Supplementary material for: Changes in the physiological activity of parenchyma cells in Dalbergia odorifera xylem and its relationship with heartwood formation
Source: BMC Plant Biol. 2023 Nov 14;23:559. doi: 10.1186/s12870-023-04592-2 (PMC10644609; doi:10.1186/s12870-023-04592-2)
Supplement: Supplementary file 1 — Supplementary Material 1 [file 12870_2023_4592_MOESM1_ESM.docx]

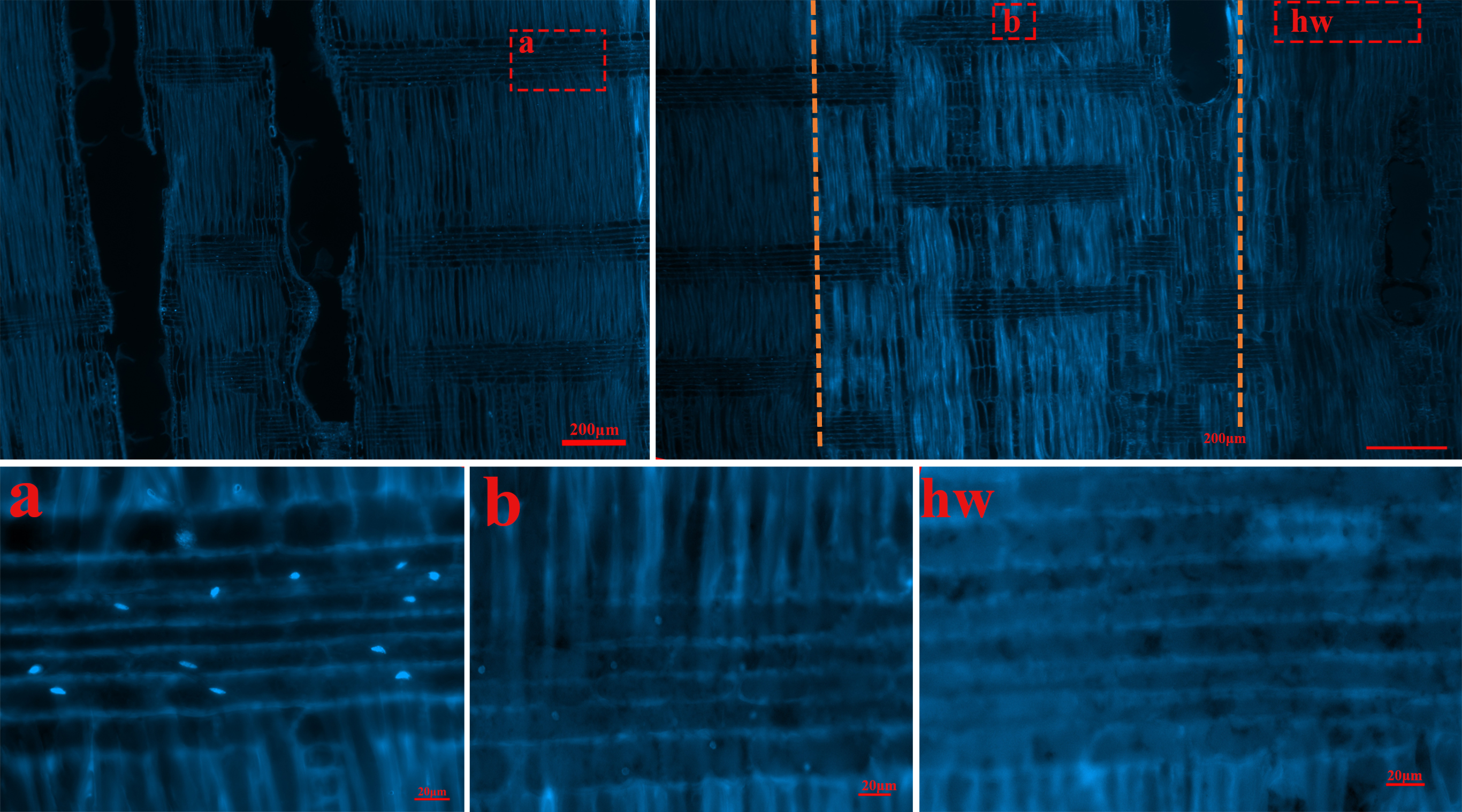


Figure S1 Morphological changes in the nucleus. Area a is located in the outer transition zone. Area b has the appearance of heartwood colour, the fluorescence of the cell wall becomes stronger compared to region a. The nucleus is not observed in region hw.
